# Adaptive Control Strategies for Interlimb Coordination in Legged Robots: A Review

**DOI:** 10.3389/fnbot.2017.00039

**Published:** 2017-08-23

**Authors:** Shinya Aoi, Poramate Manoonpong, Yuichi Ambe, Fumitoshi Matsuno, Florentin Wörgötter

**Affiliations:** ^1^Department of Aeronautics and Astronautics, Graduate School of Engineering, Kyoto University Kyoto, Japan; ^2^Embodied AI & Neurorobotics Lab, Centre for Biorobotics, Mærsk Mc-Kinney Møller Institute, University of Southern Denmark Odense, Denmark; ^3^Department of Applied Information Sciences, Graduate School of Information Sciences, Tohoku University Aoba-ku, Japan; ^4^Department of Mechanical Engineering and Science, Graduate School of Engineering, Kyoto University Kyoto, Japan; ^5^Bernstein Center for Computational Neuroscience, Third Institute of Physics, Georg-August-Universität Göttingen Göttingen, Germany

**Keywords:** legged robot, interlimb coordination, adaptation, sensorimotor interaction, central pattern generator

## Abstract

Walking animals produce adaptive interlimb coordination during locomotion in accordance with their situation. Interlimb coordination is generated through the dynamic interactions of the neural system, the musculoskeletal system, and the environment, although the underlying mechanisms remain unclear. Recently, investigations of the adaptation mechanisms of living beings have attracted attention, and bio-inspired control systems based on neurophysiological findings regarding sensorimotor interactions are being developed for legged robots. In this review, we introduce adaptive interlimb coordination for legged robots induced by various factors (locomotion speed, environmental situation, body properties, and task). In addition, we show characteristic properties of adaptive interlimb coordination, such as gait hysteresis and different time-scale adaptations. We also discuss the underlying mechanisms and control strategies to achieve adaptive interlimb coordination and the design principle for the control system of legged robots.

## 1. Introduction

Animals produce adaptive motor behaviors by skillfully manipulating their complicated and redundant musculoskeletal systems. Locomotion is an important behavior required in daily life. Gait selection in accordance with the situation, such as speed and environment, is a prominent adaptive motor function. Humans walk bipedally and use walking and running gaits. Quadruped animals use four legs and produce walking, trotting, and galloping gaits. Hexapod insects use six legs and create metachronal (wave), tetrapod, and tripod gaits as well as intermediate stepping patterns forming a continuum. These gaits, including the transitions and the intermediate stepping patterns for hexapods, are generated through the intralimb and interlimb coordination of leg movements. Intralimb coordination is the relationship between segments or joints within one leg, whereas interlimb coordination is the relationship between legs. For example, in the adaptive control of intralimb coordination, peak timings of ankle plantar flexion, knee extension, and hip extension are out of phase during the human walking gait, but they are shifted and almost in phase during the human running gait (Diedrich et al., 1998). In the adaptive control of interlimb coordination, the footfall sequence between legs changes (Muybridge, 1957), and the sequence is mainly explained by the relative phases between leg movements, because the leg movements are periodic with almost the same period for each leg (note that different frequencies between the legs have been sometimes observed in insects due to the flexibility in the stepping patterns, Pearson and Franklin, 1984). In the quadrupedal walking gait, although the left and right legs move in anti-phase, the ipsilateral front and hind legs do not. In contrast, in the quadrupedal trotting gait, the ipsilateral front and hind legs as well as the left and right legs move in anti-phase; that is, the diagonal legs move in phase (Hildebrand, 1965). Measured data analyses performed to clarify the gait mechanisms have suggested that gaits are selected based on metabolic and biomechanical factors (Margaria, 1938; Hoyt and Taylor, 1981; Farley and Taylor, 1991). However, reports on the roles of these factors in determining the gaits (Hreljac, 1993; Minetti et al., 1994; Raynor et al., 2002; Wickler et al., 2003) are conflicting, and so the underlying mechanism remains unclear.

To elucidate adaptive motor functions in animals, neurophysiological and biomechanical studies have been independently conducted. Neurophysiological studies mainly investigate the configurations and activities of the neural system, whereas biomechanical studies generally examine the functional roles of the musculoskeletal system. However, locomotion is generated through dynamic interactions among the neural system, the musculoskeletal system, and the environment. It is thus difficult to fully analyze the locomotion mechanism from a single perspective. In addition, gaits are viewed as self-organized patterns in such complex dynamical systems (Schöner et al., 1990; Diedrich et al., 1998; Griffin et al., 2004; Schilling et al., 2013a). The stability structure of gaits has been identified from the response of perturbations, especially by phase oscillators and phase response curves (Couzin-Fuchs et al., 2015; Funato et al., 2016) based on the phase reduction theory (Kuramoto, 1984). However, it is difficult to understand how the stability structure is generated due to the complex nature of interactions between the dynamic factors in locomotion. To fully elucidate the locomotion mechanism, integrated studies of neural and musculoskeletal systems are required to find the processes that create adaptive locomotor behavior.

Recently, to reveal the locomotion mechanism, legged robots have attracted attention. A robot's mechanical system with actuators, such as electric motors and pneumatic and hydraulic actuators, has been used to investigate the dynamic role of the musculoskeletal system in locomotion. The control system of the robot has been developed based on neurophysiological findings and employs various sensors, such as a touch sensor, load cell, acceleration sensor, gyro sensor, laser range scanner, and vision system. This approach allows us to emulate and investigate gait generation through dynamic interactions between the neural system, the musculoskeletal system, and the environment. In particular, central pattern generators (CPGs), which are located in the spinal cord of vertebrates and in the thoracic ganglia of invertebrates, are an important factor for elucidating the locomotion mechanism (Grillner, 1975; Orlovsky et al., 1999; MacKay-Lyons, 2002) and have aided the development of locomotion control systems of legged robots. A CPG is a group of interconnected neurons that can be activated to generate a motor pattern without the requirement of sensory feedback. The evidence that supports this hypothesis was originally shown by Brown (1911). In addition to the open-loop control function, CPGs receive sensory feedbacks to modulate motor commands. This closed-loop structure of sensory feedbacks is crucial to achieve adaptive behavior depending on the situation. Various CPG models have been proposed by using neural or oscillator networks and implemented in control legged robots [see review by Ijspeert (2008)]. For example, Taga and Shimizu (1991) and Taga (1995) conducted a pioneering study of a CPG model for human bipedal locomotion. They employed an articulated multi-link system for the body mechanical model and neural oscillators developed by Matsuoka (1985) for the CPG model. This CPG model received sensory signals of local and global information for locomotion. They demonstrated that adaptive locomotion is established through the interaction between body dynamics, oscillator dynamics, and environment; they called this “global entrainment.” Although complex and robust locomotion behavior can be achieved by purely reflexive control mechanisms (Cruse et al., 1998; Manoonpong et al., 2007; Lewinger and Quinn, 2011; Schilling et al., 2013a,b) and classical machine learning control (Bongard et al., 2006; Cully et al., 2015) instead of using CPG models, the CPG concept and modeling have had a large influence on the studies of legged robots.

In this review, we focus on the adaptive control of interlimb coordination in locomotion. We introduce adaptive interlimb coordination for animals and legged robots induced by various factors (locomotion speed, environmental situation, body properties, and task). In addition, we show characteristic properties of adaptive interlimb coordination in animals and robots, such as gait hysteresis and different time-scale adaptations. Finally, we discuss the underlying mechanisms and control strategies to achieve adaptive interlimb coordination and the design principle for the control system of legged robots.

## 2. Adaptive interlimb coordination in animals and robots

### 2.1. Speed-dependent adaptation

**Figure 1:**
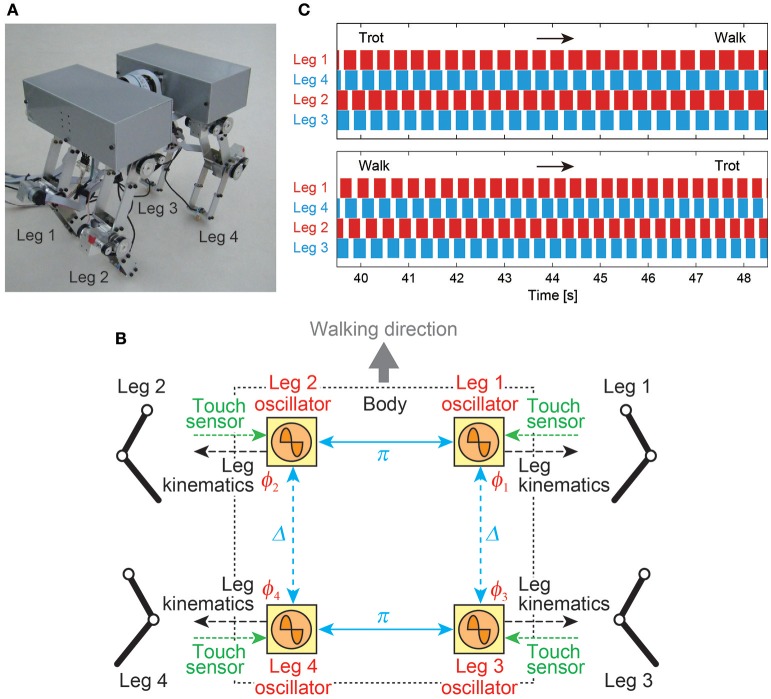
Walk–trot transition of a quadruped robot induced by changing the locomotion speed. **(A)** Quadruped robot. **(B)** CPG-based phase oscillator network with local sensory feedback of foot contact information. Left and right leg oscillators are anti-phase and front and hind leg oscillators (Δ: phase difference) are only weakly coupled. **(C)** Footfall sequences during the trot-to-walk and walk-to-trot transitions (white: swing phase, colored: stance phase). These figures were modified from Aoi et al. (2013b).

**Figure 2:**
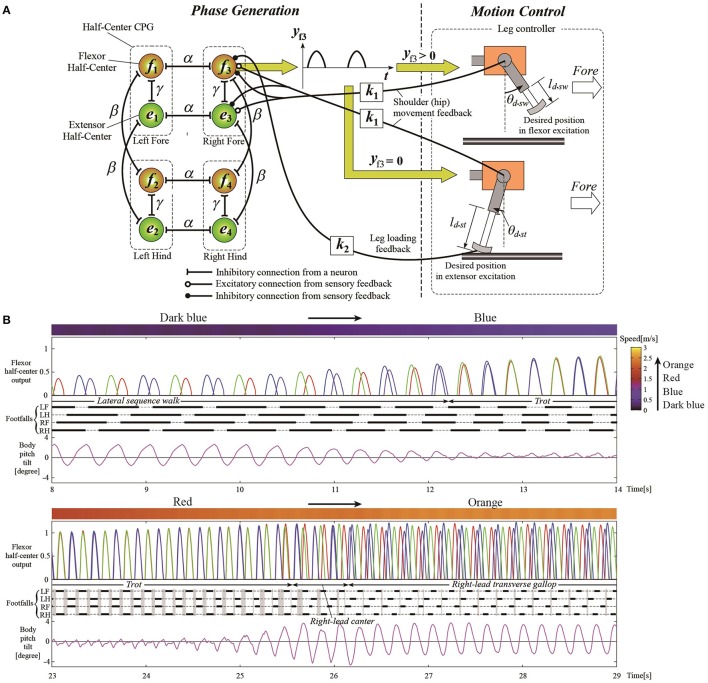
Simulation results of walk, trot, and gallop gaits with different speeds for a quadruped model. **(A)** The quadruped model and its hard-wired neural oscillator network with leg loading feedback for interlimb coordination. **(B)** The CPG outputs for controlling the legs, different emerged gaits from the interlimb coordination, and body movement around the pitch axis. The walking speeds are shown as the top meter bars (dark blue to blue and red to orange) where speed level is denoted as the color meter on the top right gradient bar. The convex curves indicate the flexor half-center outputs for left foreleg (LF, blue), left hindleg (LH, red), right foreleg (RF, green), and right hindleg (RH, purple), which lead to the swing phase. The thick lines indicate the stance phase and the thin dashed lines refer to the swing phase. These figures were modified from Fukuoka et al. (2015) with permission.

**Figure 3:**
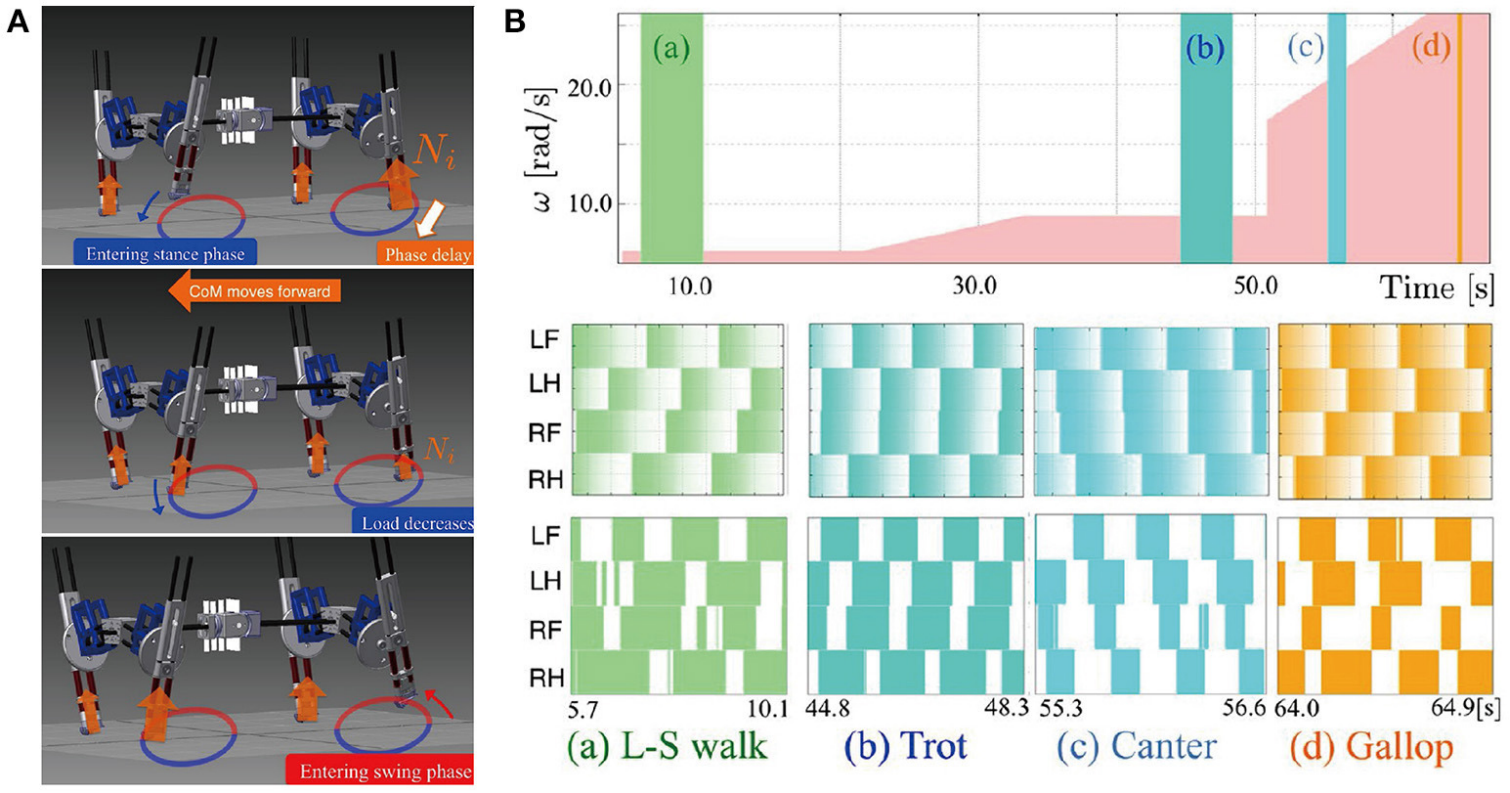
Walk-trot-gallop transitions of a quadruped robot achieved by changing its locomotion speed. **(A)** The quadruped robot with interlimb coordination generated by non-wired simple phase oscillators (CPGs) with continuous phase modulation. The oscillator phases are modulated with respect to the magnitude of local load sensing *N*_*i*_. **(B)** Walking speed and gait diagrams of different locomotion modes (walk, trot, canter, and gallop). The pink area shows the change of the treadmill speed with respect to the value of ω. The colored areas in the gait diagrams mean the stance phase, during which the sensor value *N*_*i*_ becomes greater than a threshold value. These figures were modified from Owaki and Ishiguro (2017) with permission.

The most general adaptive interlimb coordination appears when varying the locomotion speed in legged animals. This has been observed even in spinal cats on treadmills (Forssberg and Grillner, 1973; Orlovsky et al., 1999), in which the phase relationship between the legs changes and the gait varies among walking, trotting, and galloping. In reported studies, the spinal cords of cats were transected from the brain, but they still received sensory feedback through the contact between their feet and the belt. The sensory signals changed with the belt speed change, which induced their gait transitions. This result highlights the important contribution of sensorimotor interaction to adaptive interlimb coordination. Quadruped robots have achieved adaptive interlimb coordination that depends on locomotion speed by modeling spinal CPGs with local sensory feedback (Maufroy et al., 2010; Aoi et al., 2011, 2013b; Owaki et al., 2013; Fukuoka et al., 2015; Owaki and Ishiguro, 2017). This can be seen in the following examples. Figure 1 shows a quadruped robot, the control system, and the experimental results of the walk–trot transition in Aoi et al. (2013b) (this robot showed hysteresis in the gait transition, as discussed in Section 3.1). Figure 2, which is from the work by Fukuoka et al. (2015), presents quadruped gaits transitioned from a walk at slow speeds to a trot at medium speeds, and a transverse gallop at high speeds. Figure 3, which is from the work by Owaki and Ishiguro (2017), also shows spontaneous gait transitions from a lateral-sequence (L-S) walk to a trot and even to a gallop of a quadruped robot with respect to the locomotion speed without neural coupling. These robotics studies used simple neural oscillators or phase oscillators for the CPG model and produced leg motions from the oscillator phases. More specifically, one oscillator created one leg motion and the phase relationship between the oscillators determined the gait. Each oscillator phase was regulated through local sensory information of the leg, such as foot contact and leg loading, occurring only within one leg.

As an important control architecture in these robotics studies, the phase relationship between the oscillators was not predefined and the oscillators were only weakly coupled or decoupled. That is, the gait was not determined by the oscillator dynamics using strong coupling (Schöner et al., 1990; Canavier et al., 1997; Ito et al., 1998; Golubitsky et al., 1999), but by the interaction between whole-body dynamics and oscillator dynamics through local sensory feedback. The interlimb coordination was generated only in a self-organizing manner among the neural dynamics, the body dynamics, and the environment.

Similar adaptive interlimb coordination in accordance with gait speed also appears in hexapod insects, such as stick insects (Wilson, 1966; Graham, 1972; Cruse, 1990; Grabowska et al., 2012), cockroaches (Hughes, 1952; Delcomyn, 1971; Pearson, 1976; Bender et al., 2011), and flies (Strauß and Heisenberg, 1990; Wosnitza et al., 2013; Berendes et al., 2016). In particular, stick insects and flies smoothly change their interlimb coordination in accordance with gait speed (Wilson, 1966; Graham, 1972; Wosnitza et al., 2013). More specifically, the relative phases between the legs continuously change in a linear fashion for gait speed. This is similar to some mammals, including sheep, but is different from other mammals, including dogs. In mammals such as dogs, the gait transitions have relative leg phases that change suddenly in a sigmoid fashion (Alexander and Jayes, 1983). Although it is suggested that cockroaches achieve interlimb coordination mainly by the CPG itself (Fuchs et al., 2011), the CPG by itself does not produce a coordinated motor pattern for stick insect walking, because sensory feedback is important (Bässler and Wegner, 1983; Büschges et al., 1995; Büschges et al., 2008). Cruse and his colleagues proposed an artificial neural network, named Walknet, which controls leg movements based on six different rules to regulate interlimb coordination by sensory information (note that the controller of the individual leg operates without CPG). The rules were empirically derived from the behavioral experiments of stick insects [see reviews by Cruse et al. (1998), Dürr et al. (2004) and Schilling et al. (2013a)]. Three of the rules were designed by disturbing leg movements on a slippery surface. The rules changed the cycle duration of a leg based on sensory information of the neighboring legs. As a result of sensorimotor interaction, the insect models controlled by Walknet produced a continuum of locomotion patterns, such as tripod, tetrapod, and wave gaits, and intermediate stepping patterns, as observed in stick insects. In addition, the models were used for various situations, such as walking on uneven surfaces (Kindermann, 2002), leg amputation (Schilling et al., 2007), negotiating curves (Schilling et al., 2013b), and climbing over large gaps (Bläsing, 2006), and the locomotor behavior was comparable to that of stick insects. Tóth and Daun-Gruhn (2016) developed neural network models based on Hodgkin Huxley dynamics and integrated them with musculoskeletal models to explain the interlimb coordination mechanism of insects. Although their models did not produce intermediate stepping patterns as observed in stick insects (Wilson, 1966; Graham, 1972) and flies (Wosnitza et al., 2013), their results suggest that the connection between the levator-depressor neuromuscular systems of the different legs is necessary to replicate the primary features of tripod and tetrapod gaits. Ambe et al. (2013, 2015) used simple phase oscillators with local sensory feedback of foot contact information for a hexapod robot, in a manner similar to the quadruped robots mentioned above. They produced a continuum of locomotion patterns, such as metachronal and tripod gaits and intermediate stepping patterns, through embodied sensorimotor interaction, without predefining the patterns in accordance with the locomotion speed. In addition, one important aspect shown was positive velocity feedback during the stance of stick insects (Bässler, 1976). The positive velocity feedback has been tested on a robot (Schmitz et al., 2008).

Similarly, myriapods, such as centipedes, change their interlimb coordination depending on gait speed. Myriapods have a long and flexible body axis and produce body undulations when the gait speed increases (Manton, 1965). In addition to the amplitude increase of the undulations, the phase relationship between ipsilateral leg movements changes in synchronization with the body segment movements of the undulations. In Aoi et al. (2007, 2013a), a multilegged robot with six body segments and twelve legs, which use torsional springs for body axis flexibility, was developed. The robot showed body undulations through a supercritical Hopf bifurcation of straight walking by increasing the locomotion speed, and so showed dependence of body undulations on speed, as was similar to the dependence shown by centipedes.

### 2.2. Environment-dependent adaptation

The advantage of using legs in mobile motion for animals and machines is to gain high traversability even in complex environments by manipulating the foot contact positions. However, the traversability of legged robots is still far from reaching the level of animals. During locomotion, the leg motion consists of the stance phase, in which the foot is in contact with the ground, and the swing phase, in which the foot is lifted off the ground. In the stance phase, the leg supports the body against gravity and produces propulsive and decelerating forces to move the body through the interaction between the foot and the ground. Geometric properties of the ground vary. These properties include being flat terrain, sloped terrain, or irregular and rough terrain. The physical properties of the ground also change. These properties include hard and slippery ground like stone, soft ground like loose soil, and flowable and penetrable ground like sand. The interaction between the foot and the ground is crucial to create locomotion, and real-time adaptation of motor behavior is required according to the ground situation. Animals actually show adaptive interlimb coordination depending on the environmental situation. To control legged robots, it is crucial to clarify and apply the dynamical principles of animals.

**Figure 4:**
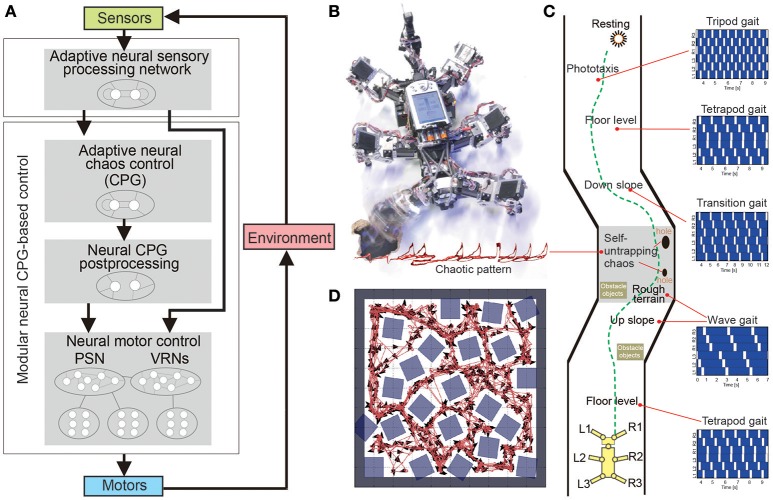
Environment-dependent adaptation of a hexapod robot under modular single CPG-based locomotion control. **(A)** Modular neural locomotion control consisting of adaptive chaos control (acting as a CPG), CPG post-processing, and neural motor control. Locomotion control can generate multiple gaits (wave, transition, tetrapod, and tripod gaits) and a chaotic pattern for locomotion on different terrains. A combination of neural control with an adaptive neural sensory processing network can generate adaptive obstacle avoidance for avoiding obstacles in a complex environment. **(B)** Hexapod robot AMOS with a chaotic movement pattern for self-untrapping (i.e., freeing itself when its leg is trapped in a hole). The red light trace recorded from an LED installed at the leg shows the chaos motion of the leg. **(C)** Walking experiment on different terrains (floor, slopes, rough terrain, and holes in the ground) where different gaits were used. The white areas in the gait diagrams indicate the swing phase and the dark blue areas refer to the stance phase. **(D)** Adaptive obstacle avoidance in a simulated complex environment with obstacles, sharp corners, and narrow passages. These figures were modified from Grinke et al. (2015) and Steingrube et al. (2010).

**Figure 5:**
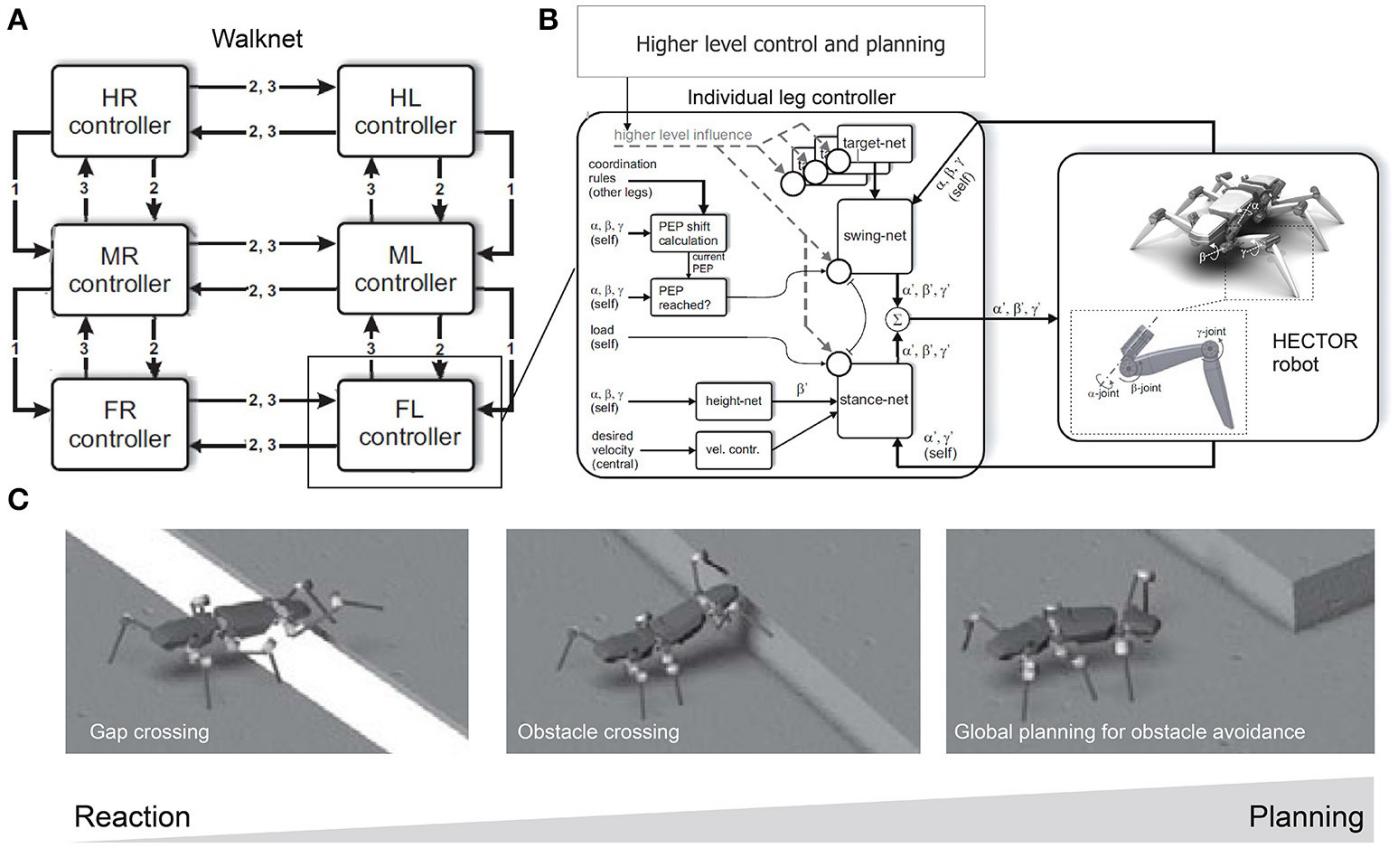
Walknet with higher level control and planning for different locomotion behavior generation of the hexapod robot HECTOR for different environments. **(A)** Bio-inspired control Walknet with interlimb coordination rules [rules 1, 2, and 3; see Schilling et al. (2013a) and Schneider et al. (2012) for details]. **(B)** Setup of an individual leg controller with higher-level control and planning. Its outputs drive the leg joints of HECTOR. **(C)** Different desired locomotion behaviors that can be generated by the control approach to deal with complex environments. These figures were modified from Schneider et al. (2012) with permission.

Manoonpong and his colleagues developed a series of modular neural CPG-based locomotion control for legged robots (Manoonpong et al., 2008, 2013; Steingrube et al., 2010; Goldschmidt et al., 2014; Xiong et al., 2014, 2015; Dasgupta et al., 2015; Grinke et al., 2015). They showed that using this control approach leads to adaptive interlimb coordination that allows the robots to deal with complex environments, such as walking over difficult terrain (Steingrube et al., 2010; Manoonpong et al., 2013; Goldschmidt et al., 2014; Xiong et al., 2014, 2015; Dasgupta et al., 2015) and avoiding obstacles in an unknown cluttered area (Manoonpong et al., 2008; Grinke et al., 2015), as observed in insects. For example, they implemented modular neural control with an adaptive chaotic CPG-based network and sensory feedback on a hexapod robot (Figures 4A,B; Steingrube et al., 2010). Due to the intrinsically chaotic dynamics of the CPG similar to that observed in certain biological CPGs (Rabinovich and Abarbanel, 1998), the dynamics were exploited to generate various walking patterns depending on the environmental condition. In their setup, the robot showed a tetrapod gait for standard walking, a wave gait for up-slope walking, a mixture gait between wave and tetrapod gaits for down-slope walking, and a tripod gait for fast walking to perform fast phototaxis (Figure 4C). However, this implementation of discrete gaits does not necessarily correspond to the situation found in insects. In addition to these multiple gaits, the chaotic dynamics especially contributed to self-untrapping of a leg from a hole in the ground (Figure 4B) and thereby enhanced foothold searching behavior. In Dasgupta et al. (2015), Goldschmidt et al. (2014), and Manoonpong et al. (2013) integrating forward models into the modular neural control enabled the robot to effectively predict its walking state in order to extend or elevate its legs during the swing and stance phases while walking on complex terrains. With this setup, the robot walked on uneven terrain by using a tetrapod gait and climbed over high obstacles as well as up a flight of stairs by using a wave gait. Moreover, it successfully crossed a large gap by using a caterpillar gait, where each left and right pair of legs moved simultaneously. In this situation, however, stick insects show more complex behavior than caterpillar coordination, which is adopted only rarely, if at all (Blaesing and Cruse, 2004). In Xiong et al. (2014), modular neural control was extended by introducing muscle models based on virtual agonist-antagonist mechanisms (VAAM), and neuromechanical control was produced to achieve leg compliance. Combining neuromechanical control with sensorimotor learning results in energy-efficient walking using different gaits with corresponding leg compliances (Xiong et al., 2015). The robot efficiently walked on different surfaces including sponge, gravel, fine gravel, and grass. For adaptation to the avoidance of obstacles in a cluttered environment, an adaptive neural sensory processing network with synaptic plasticity was introduced to the modular neural control (Grinke et al., 2015). The adaptive processing network could drive different turning behaviors with short-term robot memory. As a consequence, the robot walked around and adapted its turning behavior to avoid obstacles in different situations and to avoid sharp corners or deadlocks (Figure 4D). In addition to the modular neural control approach, Schneider et al. (2012) developed bio-inspired control, which combines Walknet (mentioned above) with higher level control and planning (Figures 5A,B), for adaptive interlimb coordination of the hexapod robot HECTOR. By using this control technique, versatile behaviors (e.g., gap crossing, obstacle crossing, and global planning to avoid or attack obstacles) can be generated to deal with complex environments (Figure 5C). Furthermore, Schilling and Cruse (2017) expanded Walknet to invent new behaviors and test them by internal simulation before using them in reality. Arena et al. (2017) proposed multilayered CPG-based locomotion control with insect inspired motor-skill learning. It can adaptively coordinate the limbs of a Drosophila-like hexapod robot for stable walking and obstacle climbing.

When horses walk up an incline (Wickler et al., 2003) or when they carry weights (Farley and Taylor, 1991), the trot-to-gallop transition speed is reduced. Hexapod insects, such as stick insects, cockroaches, and beetles, change their gait depending on the slope of the ground (Spirito and Mushrush, 1979; Pelletier and Caissie, 2001; Grabowska et al., 2012). Furthermore, while cockroaches use the tripod gait during normal walking, the gait changes to metachronal when they are tethered on a supported ball to decrease loading (Spirito and Mushrush, 1979); uphill slope and loading induce similar effects on their gaits (Tang and Macmillan, 1986). Fujiki et al. (2013a) extended the control system of a quadruped robot (Figure 1B) for a hexapod walker and showed that the gait changed between tripod and metachronal gaits through the sensorimotor interaction depending on the loading and slope angle, as observed in insects.

Fukuoka et al. (2003), Fukuoka and Kimura (2009), and Kimura et al. (2007a,b) used the neural oscillators developed by Matsuoka (1985) to control quadruped robots (Tekken series). They incorporated models of various reflexes, such as the flexor reflex, extensor reflex, and vestibulospinal reflex, based on sensory information. In addition, they modeled the tonic labyrinthine response to adjust the rolling motion to synchronize with the pitching motion. The robots produced robust locomotion over irregular terrain, such as steps and slopes, while inducing the gait transition between walking and trotting.

When the ground is flowable like sand, the leg penetrates deeply into the ground during locomotion. Consequently, the interaction with the ground to produce lift, drag, and thrust forces becomes complicated [see review by Aguilar et al. (2016)]. Li et al. (2009, 2013) used a tripod gait for a hexapod robot and produced locomotor performance similar to that in hard ground by adjusting the leg shape and leg motion with a force model of the robot moving in granular media.

Along with the adaptation to slopes, rough terrain, cluttered areas, and flowable areas, interlimb adaptation dealing with an asymmetric environmental condition has been investigated. For the asymmetric condition, split-belt treadmills have been used in studies of humans (Dietz et al., 1994; Reisman et al., 2005; Morton and Bastian, 2006), cats (Yanagihara and Kondo, 1996; Frigon et al., 2013), crayfishes (Müller and Cruse, 1991a,b), and stick insects (Bässler and Wegner, 1983; Foth and Graham, 1983). The treadmills have two parallel belts with independently controlled speeds and thus are capable of artificially creating left–right symmetric and asymmetric environments for walking (tied configuration: same speed between the belts, split-belt configuration: different speeds between the belts). Although the details are discussed later in Section 3.2, adaptive interlimb coordination has been observed in accordance with the belt speed condition. Such an adaptation appeared even in spinal cats (Forssberg et al., 1980; Frigon et al., 2013). Otoda et al. (2009) developed a sensory-driven controller without a CPG model for a two-dimensional biped robot and Fujiki et al. (2013b) used simple phase oscillators for the CPG model of a biped robot with local sensory feedback of the foot contact information, as was similarly done with the abovementioned quadruped and hexapod robots that achieved adaptive interlimb coordination (Aoi et al., 2011, 2013b; Ambe et al., 2013, 2015; Fujiki et al., 2013a). The biped robots achieved adaptive interlimb coordination on split-belt treadmills.

### 2.3. Body-dependent adaptation

Animals show adaptive motor behavior also due to changes in their body properties. As mentioned above, they change walking patterns when carrying weights or reducing their loads (Tang and Macmillan, 1986; Farley and Taylor, 1991). For fast locomotion, such as the galloping gait of cursorial quadrupeds and the undulatory walk of centipedes, the appearance of trunk and body-segment movements suggests that body flexibility is crucial for adaptive locomotion (Alexander, 1988). In Aoi et al. (2011), a quadruped walker was controlled by simple phase oscillators with local sensory foot contact information (Figure 1B) and the change in trunk flexibility induced the walk–trot transition, where walking and trotting gaits appeared for a hard trunk and a soft trunk, respectively. In Aoi et al. (2007, 2013a, 2016), a centipede-like multilegged robot showed the gait transition from straight walking to body undulatory walking through a Hopf bifurcation by changing the body axis flexibility.

**Figure 6:**
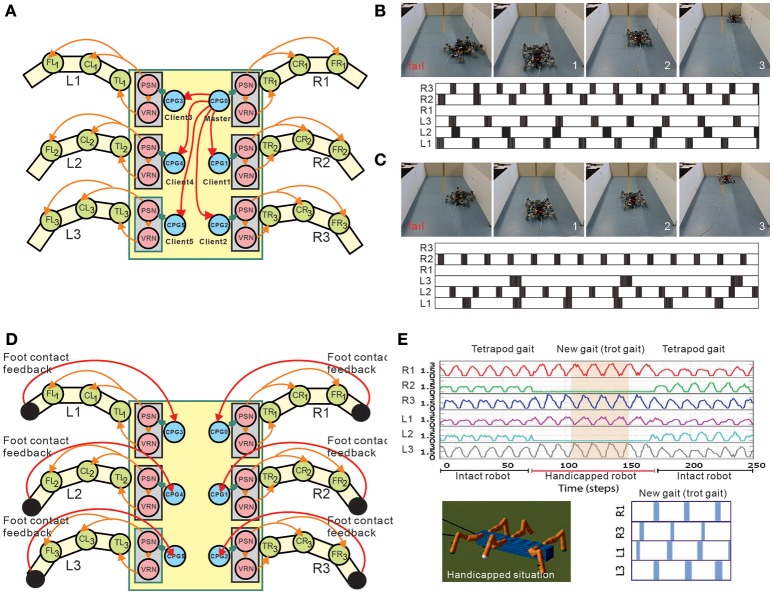
Adaptation to deal with leg malfunction of a hexapod robot under modular multiple CPG-based locomotion control. **(A)** Implementation of multiple chaotic CPGs on a hexapod robot. In the setup, the connections between CPGs are predefined. **(B,C)** Two example scenarios of real robot experiments with disabled legs. In **(B)**, the robot walked with the R1 leg disabled and in **(C)** it walked with the R1 and R3 legs disabled. For each subfigure, the upper panel shows one snapshot of a fail situation (before learning) and three snapshots of a success situation (after learning). The lower panel shows the gait (i.e., suitable leg frequencies) after learning. A black area means that the leg touched the ground, and a white area indicates that the leg was in the air. In this setup, the robot learned to find a proper combination of oscillation frequencies of different legs for malfunction compensation. **(D)** Implementation of multiple CPGs with foot contact feedback on a hexapod robot. This setup does not have predefined coordination between the CPGs. Intralimb coordination emerges from the interactions between the body dynamics and the environment through foot contact feedback of each leg. **(E)** Example of the robot experiment dealing with a temporary handicapped situation. The ground reaction forces during movement of the robot with middle legs temporarily disabled show that the robot quickly adapted to a new gait (i.e., trot gait) and was able to continue walking properly. These figures were modified from Barikhan et al. (2014) and Ren et al. (2015).

**Figure 7:**
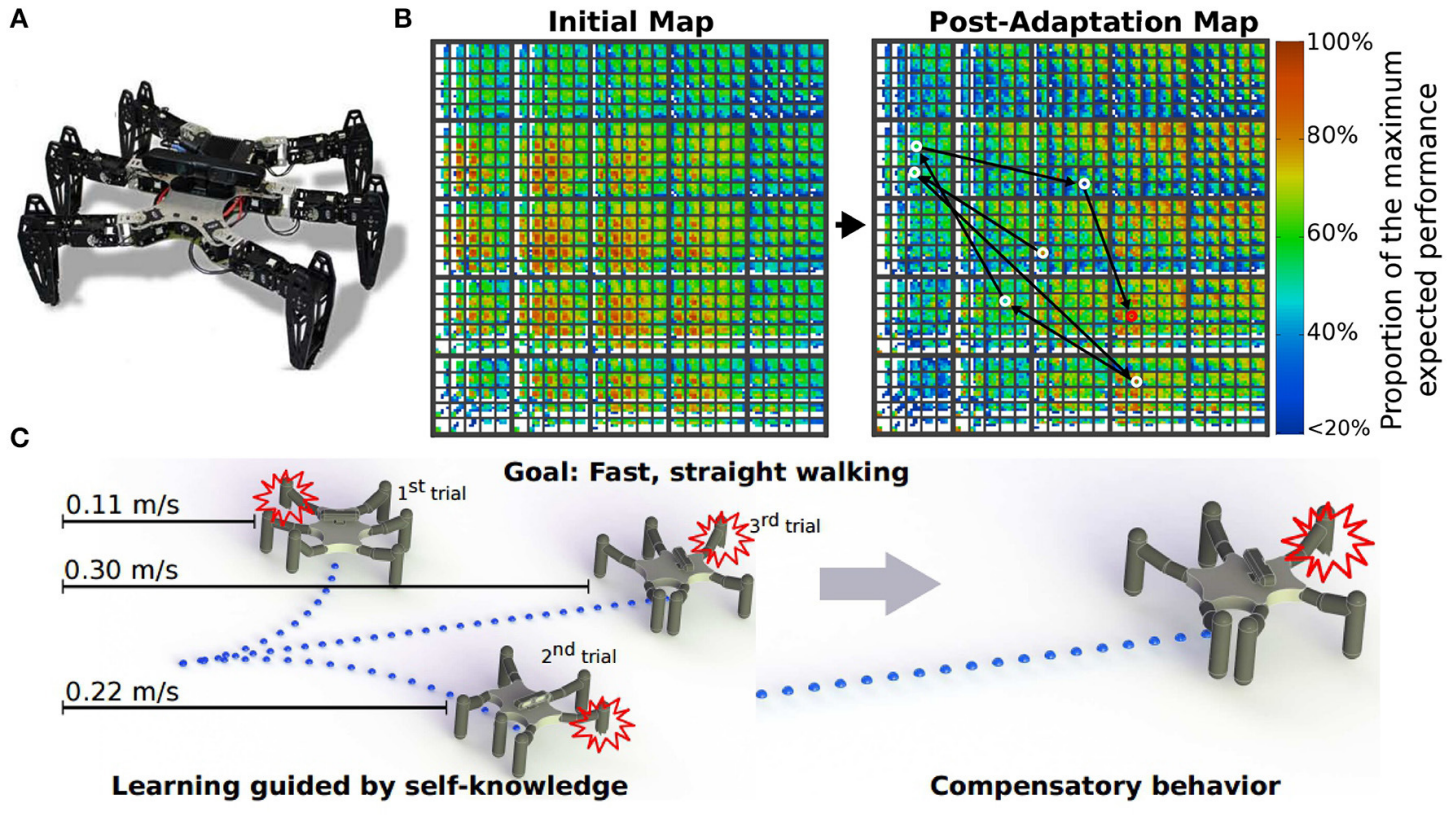
Machine learning approach for locomotion and damage recovery of a hexapod robot. **(A)** Robot setup. **(B)** Example of a behavior-performance map and its post-adaptation for damage recovery. Each colored pixel represents the highest-performing behavior discovered during map creation at that point in six-dimensional behavior space. **(C)** Robot adaptation using the trial-and-error algorithm to recover from leg damage. These figures were modified from Cully et al. (2015) with permission.

One of the advantages to using many legs for mobile motion, as in insects and myriapods, is the avoidance of losing mobility completely by leg damage due to injury and predation. Through adaptive control of interlimb coordination, even complete leg loss does not prevent walking (Grabowska et al., 2012). To clarify how interlimb coordination changes with leg loss, amputations of single legs of stick insects have been performed in order to investigate changes of the relative phases between the legs depending on which leg is amputated (Graham, 1977). Dasgupta et al. (2015) used neural CPG-based control with distributed adaptive forward models for the hexapod robot, as mentioned above, and demonstrated that the robot successfully kept walking straight with a slightly modified tetrapod gait through adaptation despite the damaged right middle leg. Ren et al. (2015) extended the chaotic CPG controller introduced above (Steingrube et al., 2010) to a controller of multiple chaotic CPGs depending on the number of legs (Figure 6A). They demonstrated that the six-legged robot (AMOSII) could continue walking by changing the interlimb coordination in accordance with the disabled leg(s) (Figures 6B,C). Walknet, which identifies the behavior of stick insects, as introduced above, was able to coordinate the movements of the remaining legs so that a six-legged walker could continue walking when some legs were amputated (Kindermann, 2002; Schilling et al., 2007). Besides these bio-inspired control approaches, Cully et al. (2015) proposed an alternative machine learning based approach consisting of two main parts: an automatically generated, pre-computed, behavior-performance map, and a trial-and-error learning algorithm (Figure 7). The behavior-performance map contains a number of interlimb coordination parameters that can generate approximately 13,000 different gaits. The trial-and-error learning algorithm is used to search for successful robot locomotion behaviors from the map with respect to robot body condition. They showed that this approach allows a hexapod robot to walk and rapidly find a walking behavior that can compensate for damage. Although all these approaches predefined interlimb connections, another approach based on the concept of emergent locomotion (i.e., walking patterns appearing as a result of stabilization in a self-organizing manner, Schilling et al., 2013a) from tight interaction between neural systems, musculoskeletal systems, and the environment has been explored for body-dependent adaptation. For example, Barikhan et al. (2014) proposed multiple decoupled neural CPGs with local sensory feedback (Figure 6D). This approach exploited the interaction between neural and body dynamics through foot contact feedback to achieve self-organized locomotion and to allow a hexapod robot to quickly adapt its locomotion to deal with morphological changes [e.g., leg damage (Figure 6E) or asymmetric leg lengths between the front and hind legs]. Tsuchiya et al. (2002) used simple phase oscillators with local sensory foot contact information to control a ten-legged robot to establish adaptive interlimb coordination, as mentioned above for quadruped and hexapod robots. The leg loss induced the change in interlimb coordination, and the change reduced the degradation of locomotion performance, such as gait speed.

### 2.4. Task-dependent adaptation

Animals often encounter a situation in which they have to change locomotor behavior. For example, when an obstacle appears in a walking path, they step over the obstacle, or turn to the right or the left to avoid collision with the obstacle (this is also related to environment adaptation). Such a task is mainly generated by modulating the leg movements, and thus adaptive control of intralimb coordination is important. However, also important is adaptive control of interlimb coordination. To step over an obstacle, the leading limb first clears the obstacle and then the trailing limb follows it. The foot of the leading limb must be raised higher than usual to avoid collision with the obstacle, and this motion delays foot contact. Especially for bipedal and quadrupedal animals, the foot of the trailing limb must be raised after foot contact of the leading limb; otherwise, the obstacle avoidance task will fail because the contralateral limb does not support the body at the onset of raising the trailing limb (Aoi et al., 2013c).

Turning behavior to change walking direction is used for various tasks, such as target pursuit (Szczecinski et al., in press) and obstacle avoidance (Figures 4D, 5C). Knops et al. (2013) controlled a mechanical model of a stick insect's middle legs by using a neural network model based on Hodgkin Huxley dynamics and produced turning behavior with two different strategies observed in stick insects walking on a slippery surface: switching the inner middle leg from forward to sideward, or from forward to backward stepping. In Aoi et al. (2016), the turning maneuverability of a centipede-like multilegged robot was enhanced via straight walk instability induced by the Hopf bifurcation by changing the body axis flexibility. Although arthropods with sprawling legs have a low center of mass and thus cannot effectively lean, mammals with erect legs have a high center of mass and can use body leaning to help turning. The relative phase between legs in human turning shifts from anti-phase due to the left–right asymmetry of the turning movement (Courtine and Schieppati, 2003). In Aoi and Tsuchiya (2007), simple phase oscillators with local sensory feedback about foot contact information were used for turn walking of a biped robot, as was used for walking on a split-belt treadmill. The relative phase between legs shifted depending on the turning radius to compensate for the left–right asymmetry induced by body leaning; this shift allowed the robot to achieve high turning performance.

**Figure 8:**
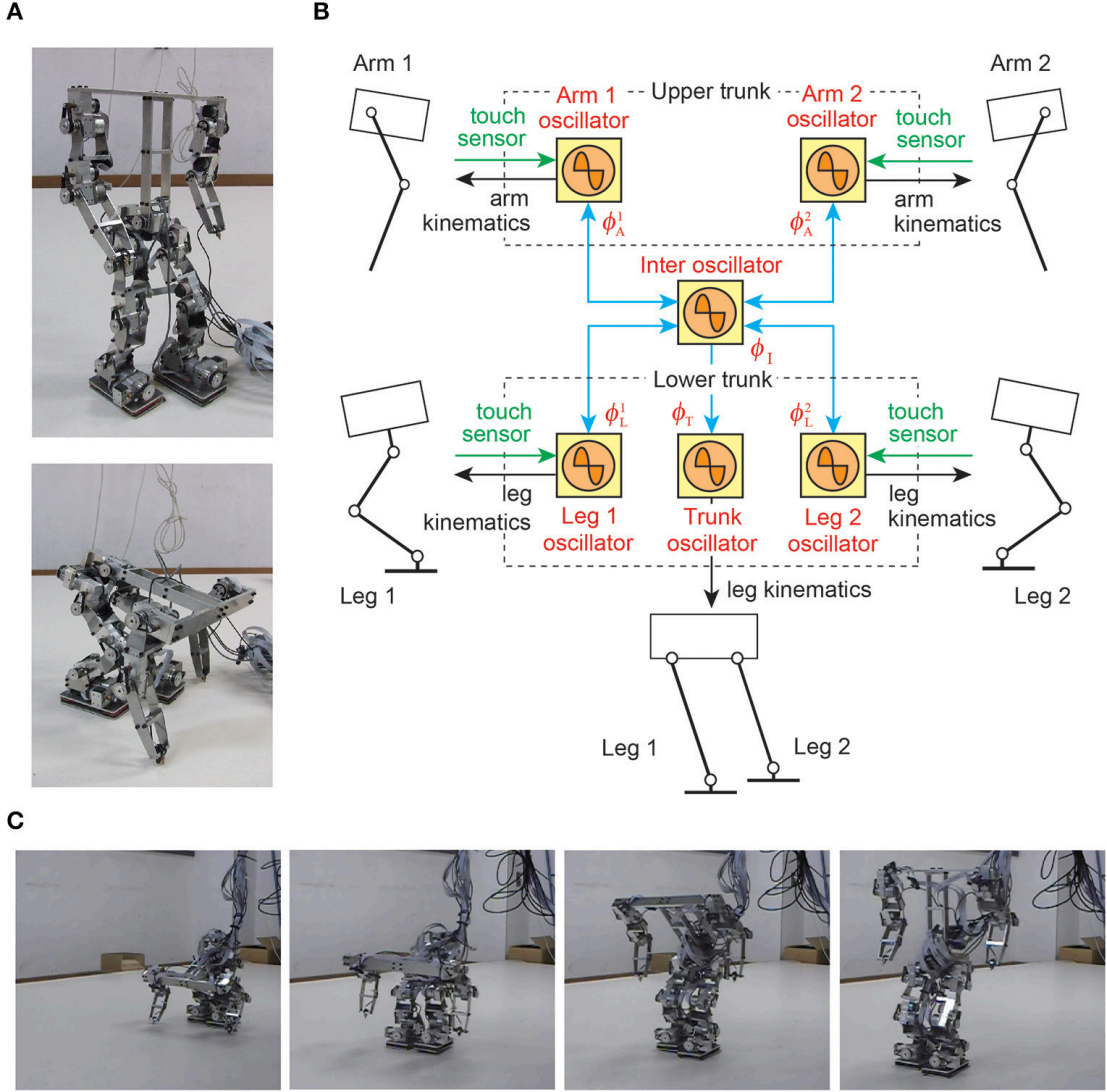
Gait transition of a biped robot from quadrupedal to upright and bipedal gait. **(A)** Biped robot. **(B)** Phase oscillator network with local sensory foot contact information for the biped robot. **(C)** Gait transition experiment. These figures were modified from Aoi et al. (2012).

The transition from quadrupedal gait to upright and bipedal gait is a challenging task for legged robots, because it requires drastic changes in locomotor movements (Asa et al., 2009; Aoi et al., 2012; Kobayashi et al., 2015). In particular, because the robot has to raise its trunk so that the arms leave the ground, an adequate relationship between the supporting limb locations and the center of mass location is important. That is, adequate interlimb and trunk coordination is crucial; otherwise, the robot easily falls over. In Aoi et al. (2012), simple phase oscillators with sensory regulation by ground contact information of the arms and legs were used for a biped robot (Figures 8A,B). The controller was extended based on the concept of kinematic synergy (Freitas et al., 2006; Ivanenko et al., 2007; Latash, 2008; Funato et al., 2010) to change the robot movements for gait transition and allowed the robot to successfully change the gait from quadrupedal to upright and bipedal (Figure 8C).

**Figure 9:**
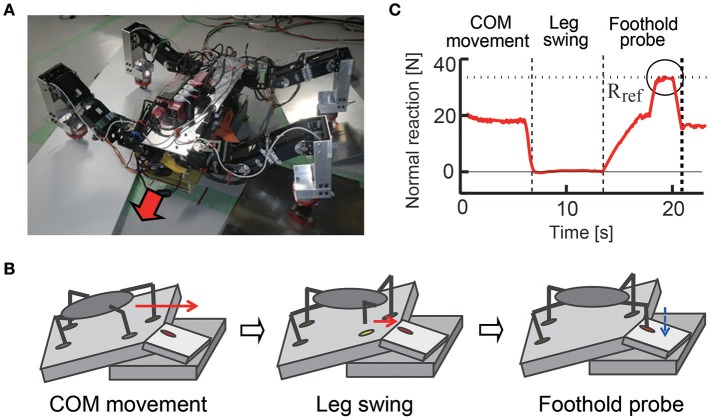
Walking on fragile irregular terrain. **(A)** Quadruped robot with load sensors on feet. **(B)** Process to find footfold condition for fore right leg. The robot moves the center of mass by standing on all four legs, then swings a leg, and probes the foothold by applying force gradually. The robot repeats this process for each of the four legs. **(C)** Time response of normal reaction force of the leg in experiments. The robot applies force over a reference value *R*_ref_ in the grope phase to ensure that the ground is solid enough for walking, but the robot never applies force over *R*_ref_ in the other phases. These figures were modified from Ambe and Matsuno (2016).

Legged robots are useful for search and rescue missions. In this case, the ground is not only irregular but also fragile, like an area with scattered debris and collapsed buildings, on which surfaces may collapse when put under external forces, such as the pressure from a robot's leg. It is important to check the ground condition in such situations by using haptic information of the legs to secure stable walking. In Ambe and Matsuno (2016), a control mechanism with haptic sensory feedback for terrain determination was proposed. With the control mechanism, a quadruped robot can sense whether the foothold is stable through its force sensor when it puts its leg on the ground. In addition, this mechanism produces adequate interlimb coordination so that the robot never stumbles, even if the foothold collapses in the probe motion. As a result, the robot can effectively walk on unstable terrain and avoid stumbling and causing a large collapse of the surrounding area (Figure 9). Other methods have also been proposed to estimate fragile and slippery footholds based on haptic feedbacks and image information (Tokuda et al., 2003; Hoepflinger et al., 2010, 2013).

## 3. Characteristic properties of adaptive interlimb coordination

### 3.1. Hysteresis in gait transition

**Figure 10:**
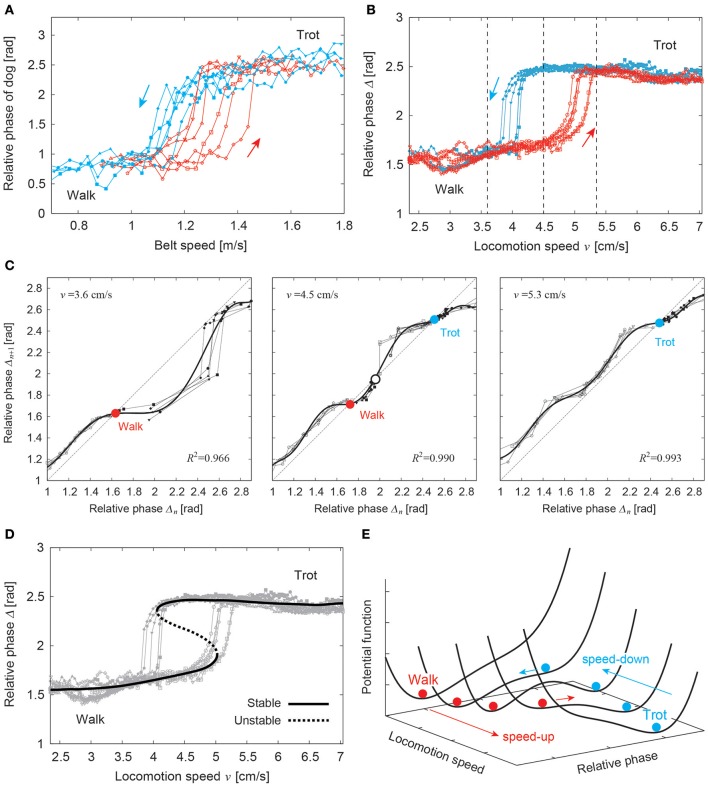
Hysteresis in the walk–trot transition. **(A)** Relative phases of ipsilateral legs of a dog for walk-to-trot and trot-to-walk transitions induced by changing the belt speed. **(B)** Relative phases of a quadruped robot for walk-to-trot and trot-to-walk transitions induced by changing the locomotion speed. The three speeds indicated by vertical dotted lines are used in **(C)**. **(C)** Stability analyses using return maps for the relative phases at three speeds. The bold lines are approximated polynomial functions of the return maps. **(D)** Estimated stable and unstable relative phases from the stability analyses showing two saddle-node bifurcations. **(E)** Possible potential function that shows hysteresis. These figures were modified from Aoi et al. (2013b).

As discussed in Section 2.1, animals change their walking patterns depending on their locomotion speed. In general, locomotion speed has a large sudden change at gait transition in overground walking. However, using treadmills, which can control gait speed, we can investigate the speed-dependent gait transition mechanism by smoothly and continuously changing the belt speed of the treadmills. It has been reported in humans and some quadruped animals that the gait changes at different speeds depending on whether the speed is increasing or decreasing, and that a speed range exists in which different gaits are used. In other words, gait transitions may exhibit hysteresis (Diedrich et al., 1998; Heglund and Taylor, 1998; Raynor et al., 2002; Griffin et al., 2004). Figure 10A shows the relative phase between the right front and hind legs of a dog walking on a treadmill for walk-to-trot and trot-to-walk transitions induced by changing the belt speed (Aoi et al., 2013b). This figure shows hysteresis in the walk–trot transition. Such a phenomenon is difficult to explain by triggering the gait transition based on metabolic and biomechanical factors. The dynamical system approach might provide useful insights into such a gait transition mechanism (Diedrich et al., 1998).

Quadruped robots controlled by simple phase oscillators with local sensory foot contact information, as introduced in Section 2.1, showed hysteresis in the walk–trot transition induced by changing the locomotion speed (Figure 10B; Aoi et al., 2011, 2013b). Because walking and trotting gaits are mainly distinguished by the relative phases of the ipsilateral legs, a stability analysis using the return maps of the relative phases clarified the stability structure of the gaits. Figure 10C shows the return maps obtained at three different speeds. While only one stable relative phase exists in the left and right figures, two stable and one unstable relative phases exist in the middle figure. The stable and unstable relative phases explain that hysteresis is generated through two saddle-node bifurcations induced by changing the locomotion speed (Figure 10D). From this result, a potential function is derived, as shown in Figure 10E. It suggests that gait transition is explained by switching the stability of self-organized patterns in the complex dynamical system.

Gait transition hysteresis also appears in other legged robots controlled by CPG models with sensory feedback, e.g., in the walk–run transition of a biped model (Taga and Shimizu, 1991) and the metachronal–tripod gait transition of hexapod robots (Kimura et al., 1993; Fujiki et al., 2013a; note that insects do not clearly show abrupt transitions, but a continuum of locomotion patterns).

### 3.2. Two different time-scale adaptations

When the environment suddenly changes, locomotor behavior is rapidly modulated to adapt to the environmental variation and successively shows gradual regulation for gaining a new locomotor pattern. This behavior suggests that motor learning occurs. This has been observed in interlimb coordination during locomotion. In particular, the split-belt treadmill walking mentioned above is a good example.

**Figure 11:**
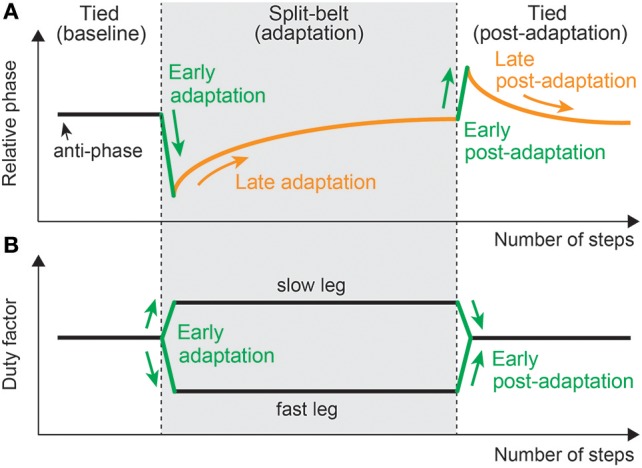
Changes in locomotor factors during human split-belt treadmill walking, where the belt speed condition changes from the tied to the split-belt configuration (adaptation period) and returns to the tied configuration (post-adaptation period). **(A)** Relative phase between legs as one of the interlimb coordination factors. This shows both early and late adaptations when the environmental change occurs. When the environment is returned, after-effects appear. **(B)** Duty factor of the legs as one of the intralimb coordination factors. This shows only early adaptation when the environment changes. These figures were modified from Fujiki et al. (2015).

The regulation of motor behavior in split-belt treadmill walking appears in various locomotor factors. However, the factors related to interlimb coordination, such as the relative phase between the legs, step length, and center of pressure profile, and those related to intralimb coordination, such as the duty factor and stride length, show different trends (Figure 11). A sudden environmental variation rapidly changes the factors; this is called “early adaptation”. Although the intralimb coordination factors do not show further change, the interlimb coordination factors tend to gradually return to their original state after early adaptation; this is called “late adaptation”. This means that interlimb coordination has two types of adaptations with different time scales. Furthermore, when the environment is returned its original state, the interlimb coordination factors move in the opposite direction to the early adaptation, which shows the after-effects.

**Figure 12:**
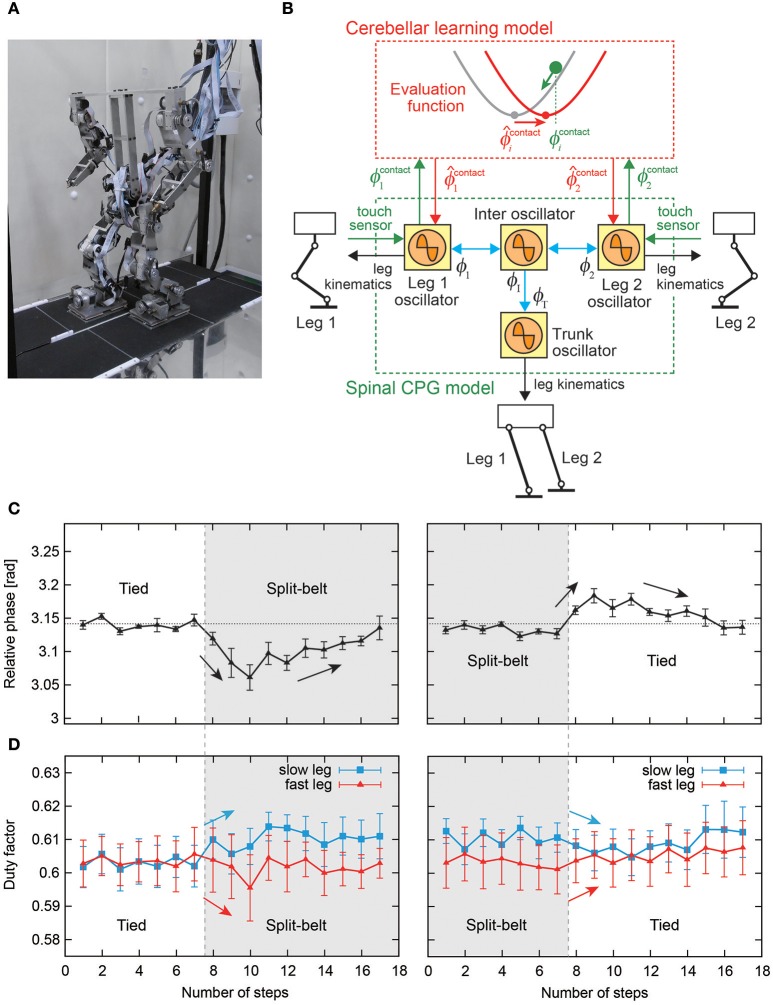
Split-belt treadmill walking experiment of a biped robot. **(A)** Biped robot and split-belt treadmill. **(B)** Spinal CPG and cerebellar learning models using simple phase oscillators with sensory reflex by local foot contact information and modulation of foot contact timing through the evaluation of prediction error. **(C)** Relative phase between legs of the biped robot. **(D)** Duty factor of legs of the biped robot. These figures were modified from Fujiki et al. (2015).

Rapid changes in the locomotor factors have been observed during split-belt treadmill walking of spinal cats (Forssberg et al., 1980; Frigon et al., 2013). These rapid changes suggest that early adaptation is induced by sensorimotor interaction in the spinal cord. On the other hand, humans with cerebellar damage do not show late adaptation or after-effects during split-belt treadmill walking, and it appears that the cerebellum contributes to late adaptation and the after-effects (Morton and Bastian, 2006; although split-belt experiments have been performed for arthropods (Bässler and Wegner, 1983; Foth and Graham, 1983; Müller and Cruse, 1991a,b), the results showed that they do not necessarily need learning, which may underestimate their adaptation ability). Otoda et al. (2009) modeled the stepping reflex to modulate the touchdown angle of the swing leg and introduced the adjustment of proportional control gain at the hip joint of the stance leg as the cerebellar function producing split-belt treadmill walking of a two-dimensional biped robot, although they did not use a CPG model with adaptation. In contrast, Fujiki et al. (2015) incorporated a cerebellar learning model into the spinal CPG model (Figure 12B). The CPG model was composed of simple phase oscillators with sensory reflex by local foot contact information and was used in Fujiki et al. (2013b) as mentioned above. The learning model modulated the foot contact timing of each leg through the evaluation of prediction error by using the local sensory foot contact information of each leg. Biped robot experiments on a split-belt treadmill (Figure 12A) showed adaptive intralimb and interlimb coordination (Figures 12C,D). In particular, despite the lack of direct interlimb coordination control, early and late adaptations and after-effects were observed in interlimb coordination, and showed strong similarities to those observed in humans.

**Figure 13:**
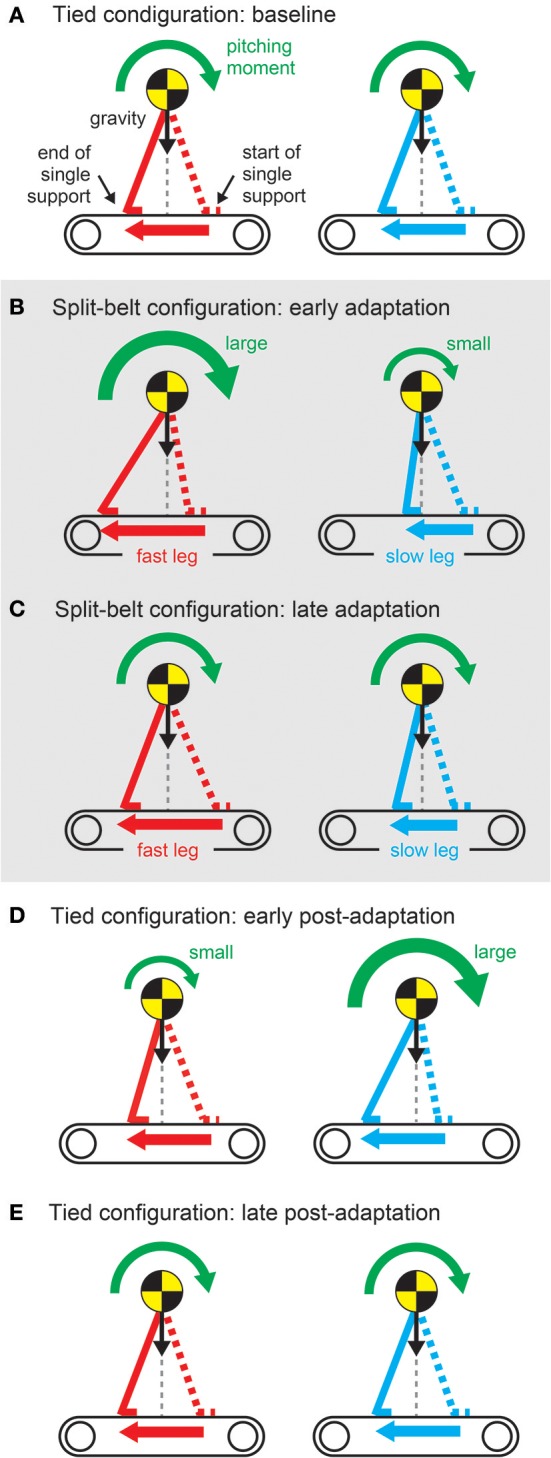
Pitching moment change due to belt-speed change **(A–E)** through sensory reflex and learning regarding foot contact timing. These figures were modified from Fujiki et al. (2015).

Rapid modulation by the sensory reflex model and gradual modulation by the learning model changed the pitching moment depending on the belt speed condition through the body dynamics of the robot (Figure 13). The pitching moment change induced spatiotemporal modification of the robot movements and altered various locomotor factors. The sensory reflex model secured the ability to continue walking against the environmental change, and the cerebellar learning model modulated the robot movements under those conditions to make walking smoother and more efficient through optimization (minimization of prediction error of foot contact timing). For simple human behaviors, such as arm reaching movements, learning models that aim to minimize jerk or torque-change have been proposed (Flash and Hogan, 1985; Uno et al., 1989). However, for human locomotion, it remains unclear what factors are predicted and how to facilitate the learning. This is partly because locomotion is a whole-body movement through limb movement and posture controls, and is governed by complicated dynamics including foot contact and lift off, which change the physical constraints. Robot experiments with neurophysiologically inspired control models are useful for examining potential control models through the comparison of results obtained from human measured data and clarification of dynamical mechanisms.

## 4. Key factors and mechanisms for adaptive interlimb coordination

In the previous sections, we presented adaptive interlimb coordination of animals and legged robots to deal with different locomotion speeds, environmental situations, body properties, and tasks. Here, we discuss key factors and mechanisms underlying the adaptive control of interlimb coordination.

One of the key mechanisms is the CPGs, which are located in the spinal cord of vertebrates and in the thoracic ganglia of invertebrates. Except for the anti-phase activity of antagonistic excitatory motoneurones, no feature of the pilocarpine-induced rhythm appears to correspond to any motor output observed in stick insects (Büschges et al., 1995). However, neurophysiological studies have revealed that CPGs are important for locomotion (Grillner, 1975; Orlovsky et al., 1999; MacKay-Lyons, 2002). A CPG is a group of interconnected neurons that can be activated to generate a motor pattern without the requirement of sensory feedback. As described in Ijspeert (2008), various CPG models with different levels of complexity have been proposed, from detailed biophysical models using Hodgkin-Huxley neurons (Traven et al., 1993; Cataldo et al., 2006; Holmes et al., 2006; Bungay and Campbell, 2009) and connectionist models using leaky-integrator neurons or integrate-and-fire neurons (Buchanan, 1992; Arena, 2000) to abstract models using coupled oscillators (Ijspeert et al., 2007; Chung and Slotine, 2010; Yu et al., 2014). Although some robot studies have shown that complex insect behavior, such as continuous gait transition, walking over irregular ground including a large gap, and curve walking with an irregular step pattern, can be replicated without CPG models (Cruse et al., 1998; Lewinger and Quinn, 2011; Schilling et al., 2013a,b), these CPG models have improved locomotion control of legged robots, such as the control of speed (Ijspeert, 2008) and robustness against sensory noise as well as sensory failure (Di Canio et al., 2016). In particular, key issues for controlling legged robots are design of feedforward and feedback controllers and integration of these controllers. The CPG models give us useful ideas for the design and integration so that the integrated controller works in a biologically plausible fashion (comparison between the controllers with and without CPG models would be useful to find the contribution of the CPG models).

Most research has employed abstract CPG models with hardwired connections to motor units for generating different basic locomotor behaviors, such as walking and swimming. Switching between different gaits or locomotion modes can be done by using simple external input signals (Kirchner et al., 2002; Ijspeert et al., 2007; Manoonpong et al., 2008). Though CPGs acting as open-loop control are the key for production of basic rhythmic locomotion, sensory feedback is a very important factor needed for adaptations to different speeds, environments, bodies, and tasks, as described in previous sections for adaptive interlimb coordination. Combining CPGs with sensory feedback results in closed-loop control with adaptability. For robotic implementation, different sensory feedback affecting CPG activities includes proprioceptive feedback (e.g., joint/leg movement and force) and exteroceptive feedback (e.g., foot contact and vision). Such feedback can modulate the frequency, phase, and magnitude of CPG activities [see review by Buschmann et al. (2015)].

Frequency modulation (also known as entrainment, Buchli et al., 2006) uses feedback information to adapt the frequency of the CPG so that the frequencies of the feedback and the CPG are synchronized (Nachstedt et al., 2017). Usually, joint angle feedback is used for this process in robotics studies (Endo et al., 2004; Buchli and Ijspeert, 2008; Di Canio et al., 2016) and frequency modulation has been mainly employed for adaptations of locomotion speed (Harischandra et al., 2011; Di Canio et al., 2016) and body change (Ren et al., 2015). In contrast, phase modulation typically uses foot contact and foot loading feedbacks to adjust the phase of CPGs to regulate the swing and stance phase durations, depending on the situation. In particular, the phase resetting mechanism, which has often been used for phase modulation in legged robots, was developed from the phase shift and rhythm resetting behaviors by the tactile sensor feedback in cats (Conway et al., 1987; Duysens, 1997; Schomburg et al., 1998; Rybak et al., 2006; Frigon et al., 2010) and stick insects (Büschges, 1995; Bässler and Büschges, 1998). The functional role of phase resetting has been investigated by the integration with musculoskeletal models and muscle synergy hypothesis (Aoi et al., 2010, 2013c; Aoi and Funato, 2016), and the control strategy was implemented in legged robots and helped to improved the robustness of their walking (Tsuchiya et al., 2002; Aoi and Tsuchiya, 2005, 2007; Nomura et al., 2009; Aoi et al., 2011, 2012, 2013b; Ambe et al., 2013, 2015; Fujiki et al., 2013a,b, 2015). Phase modulation has also been widely used for different adaptations including locomotion speed (Tsuchiya et al., 2002; Aoi et al., 2011, 2013b; Ambe et al., 2013, 2015; Fujiki et al., 2013a; Owaki et al., 2013; Fukuoka et al., 2015; Owaki and Ishiguro, 2017), environmental condition (Aoi and Tsuchiya, 2005; Aoi et al., 2010; Fujiki et al., 2013a,b, 2015), body properties (Tsuchiya et al., 2002; Aoi et al., 2011; Fujiki et al., 2013a; Owaki et al., 2013; Barikhan et al., 2014), and task (Aoi and Tsuchiya, 2007; Aoi et al., 2012, 2013c). Magnitude modulation uses different types of feedback, such as force and vision, to regulate the magnitude of the CPG. This regulation is indirectly achieved through premotor neuron networks (Buschmann et al., 2015). Goldschmidt et al. (2014) and Grinke et al. (2015) employed this strategy by using visual feedback for environment-dependent adaptation, such as hexapod robots climbing over an obstacle or turning away from it.

One can also achieve adaptive interlimb coordination by integrating these CPG modulation techniques with other bio-inspired approaches, such as adaptive muscle stiffness control (Xiong et al., 2015). Manoonpong et al. (2013) showed that bio-inspired forward models that translate motor commands or efference copies into expected sensory feedback are important components for environment-dependent adaptation, i.e., walking on different terrains. By using a split-belt treadmill, Fujiki et al. (2015) showed that cerebellar learning models to regulate motor commands while minimizing the prediction error are also important for environment-dependent adaptation. Table 1 roughly categorizes the key mechanisms that have been used for different adaptations.

**Table 1:** Key mechanisms used for different adaptations.

**Adaptation**	**Key mechanism**
Speed-dependent	CF, CP, PF
Environment-dependent	CF, CP, PF, CP+LM, CPG+FM, CPG+FM+MS
Body-dependent	CF, CP, PF, ML
Task-dependent	CP, CM, PF

*CPG, Central pattern generator; CF, CPG frequency modulation; CP, CPG phase modulation; CM, CPG magnitude modulation; PF, Pure feedback; FM, Forward model; LM, Learning model; MS, Muscle stiffness adaptation; ML, Other machine learning approaches*.

In addition to these bio-inspired key factors (CPGs, sensory feedbacks, forward model, learning model, and muscle stiffness), which are usually applied to independent control of individual legs or joints, most of the studies explicitly design complete interlimb connections to obtain the desired locomotor behaviors. This results in limitations of adaptive and flexible interlimb coordination (e.g., Kirchner et al., 2002; Ijspeert et al., 2007; Harischandra et al., 2011; Manoonpong et al., 2013; Ren et al., 2015). To overcome these limitations, a proposed alternative paradigm achieves interlimb coordination by local sensing, body-environment interactions, and weakly-coupled or decoupled CPGs (Tsuchiya et al., 2002; Aoi et al., 2011, 2013b; Shim and Husbands, 2012; Ambe et al., 2013, 2015; Fujiki et al., 2013a; Owaki et al., 2013; Barikhan et al., 2014; Owaki and Ishiguro, 2017), rather than by predefined interlimb connections. Although the proposed paradigm leads to high flexibility and adaptability in interlimb coordination, it sometimes encounters unstable locomotion. Phase resetting, which modulates the CPG phase based on the sensory reflex, as mentioned above, is one of the solutions to obtain flexible and adaptive interlimb coordination while keeping stability in locomotion (Tsuchiya et al., 2002; Aoi and Tsuchiya, 2007; Aoi et al., 2011, 2012, 2013b,c; Ambe et al., 2013, 2015; Fujiki et al., 2013a,b, 2015). However, this uses only phase modulation and has limitations. Thus, one future research study in this direction is to find a method that can autonomously form the plastic connections for stable but still flexible and adaptive locomotion. Furthermore, the interactions of CPGs, sensory feedback, body dynamics, forward model, learning model, and muscle stiffness for highly adaptive, robust, and energy-efficient locomotion remain to be explored.

## 5. Conclusion

Although walking animals create adaptive locomotor behavior by skillfully manipulating their complicated and redundant musculoskeletal systems, the underlying mechanisms are still unclear. Designing the control architecture for legged robots to autonomously achieve such adaptability is still a challenge. Although some legged robots produced adaptive locomotor behaviors by purely engineering approaches without inspiration from biological systems, neurophysiological findings such as CPG organizations and sensorimotor interactions are useful for designing the control system of legged robots. Robot experiments with CPG models and sensory feedbacks are insightful from a dynamic viewpoint for understanding gait generation and adaptation in a self-organizing manner among neural dynamics, body dynamics, and environment. In this review, we showed adaptive interlimb coordination in the locomotion of animals and legged robots induced by various factors, such as locomotion speed, environmental situation, body properties, and tasks. We also showed characteristic properties of adaptive interlimb coordination, such as gait hysteresis and different time-scale adaptations. Legged robots are becoming a valuable tool for understanding the locomotion mechanism including interlimb coordination. In the future, together with the improvement of robotics systems, such as actuators and sensors, it will be important to enhance biological plausibility and feasibility by the integration with sophisticated models of neural and musculoskeletal systems, such as the Hodgkin-Huxley model and the muscle-tendon unit model, and to extract dynamical features by integration with simple models, such as the template model (Full and Koditschek, 1999; Holmes et al., 2006). Furthermore, it will also be important to further improve and develop analytical methods, such as phase reduction theory (Kuramoto, 1984) and synergy analysis (Ivanenko et al., 2004; Latash, 2008), to clarify essential factors from multiple and redundant data.

## Author contributions

SA and PM contributed to the conception and design of the paper. SA, PM, and YA reviewed the relevant literature and wrote the paper. FM and FW revised the paper critically for important intellectual content. All authors approved the paper for publication.

### Conflict of interest statement

The authors declare that the research was conducted in the absence of any commercial or financial relationships that could be construed as a potential conflict of interest.
